# Inhibition of autophagy enhances anticancer effects of bevacizumab in hepatocarcinoma

**DOI:** 10.1007/s00109-012-0966-0

**Published:** 2012-10-10

**Authors:** Xian-ling Guo, Ding Li, Kai Sun, Jin Wang, Yan Liu, Jian-rui Song, Qiu-dong Zhao, Shan-shan Zhang, Wei-jie Deng, Xue Zhao, Meng-chao Wu, Li-xin Wei

**Affiliations:** 1 Tumor Immunology and Gene Therapy Center, Eastern Hepatobiliary Surgery Hospital, The Second Military Medical University, 225 Changhai Road, Shanghai, 200438 People’s Republic of China; 2 Hang Zhou Sanitarium of Navy, Zhejiang, People’s Republic of China; 3 Department of Oncology, Chang Zheng Hospital, The Second Military Medical University, Shanghai, People’s Republic of China

**Keywords:** Hepatocarcinoma, Antiangiogenesis, Autophagy, Metabolic stress, Apoptosis

## Abstract

Angiogenesis inhibitors have long been considered desirable anticancer agents. However, it was found that many tumors could develop resistance to antiangiogenesis inhibitors. Antiangiogenic therapy results in metabolic stress. Autophagy is an important survival mechanism in cancer cells under metabolic stress; however, it remains unknown if autophagy contributes to antiangiogenesis resistance. In this study, we reported that bevacizumab treatment reduced the development of new blood vessels and inhibited cell growth in xenografts of hepatocellular carcinoma (HCC) tumors. Bevacizumab treatment also upregulated expression of the autophagy-related genes (Beclin1 and LC3) and increased autophagosome formation. Our in vitro studies demonstrated that autophagy inhibition significantly increased apoptosis of HCC cells during nutrient starvation or hypoxia. In addition, the combined treatment of an autophagy inhibitor and bevacizumab markedly inhibited the tumor growth of HCC xenografts, led to enhanced apoptosis, and impaired the proliferation of tumor cells compared with treatment with either drug alone. Furthermore, autophagy inhibition led to enhanced reactive oxygen species (ROS) generation in HCC cells exposed to nutrient starvation or hypoxia in vitro and increased DNA oxidative damage in vivo. Antioxidants reduced nutrient starvation or the hypoxia-induced cell death of HCC cells after autophagy inhibition. Our results suggest that autophagy modulates ROS generation and contributes to cell survival under metabolic stress. Therefore, autophagy inhibition may be a novel way of increasing the efficicacy of antiangiogenic agents in the treatment of HCC.

**Electronic supplementary material:**

The online version of this article (doi:10.1007/s00109-012-0966-0) contains supplementary material, which is available to authorized users.

## Introduction

Hepatocarcinoma (HCC) is a hypervascular tumor characterized by neovascularization. Its growth relies on the formation of new blood vessels [1]. Vascular endothelial growth factor (VEGF) expression is upregulated in HCC compared with the cirrhotic or normal liver [2, 3]. These findings strongly suggest that antiangiogenic strategy may be therapeutically beneficial in the management of HCC. The effects of several antiangiogenic agents, including bevacizumab, have been evaluated in preclinical studies. Bevacizumab is a recombinant, humanized monoclonal antibody that binds VEGF with high affinity and has been approved by the FDA for treatment of certain late-stage cancers [4, 5]. Several studies have explored the clinical efficacy of bevacizumab in patients with advanced HCC; however, its therapeutic effects have not been satisfied [6].

Autophagy is an evolutionarily conserved mechanism that exists in all eukaryotes from yeast to mammals [7]. During autophagy, a double membrane, known as the isolation membrane, wraps around portions of the cytoplasm and intracellular organelles to form a double-membrane vesicle called autophagosome. Upon maturation, autophagosomes fuse with lysosomes to form autolysosomes and are degraded by lysosomal proteases [8, 9]. In addition to its homeostatic role, the autophagic process allows cells to survive nutrient stress, by providing amino acids and fatty acids to maintain metabolic levels. The genes that regulate autophagy were first identified in yeasts, many of which have homologues in higher eukaryotes [10]. Among them, LC3 [11] and Beclin1 [12] are widely used as markers of autophagy.

Antiangiogenic therapy leads to nutrient deprivation and oxygen stress in solid tumors; autophagy helps cells cope with these unfavorable conditions. Therfore, we hypothesized that autophagy inhibition could enhance the therapeutic efficacy of antiangiogenic treatments. To test this, we investigated the effect of bevacizumab on autophagy and determined if autophage inhibition could enhance the sensitivity of hepatocarcinoma to bevacizumab treament.

## Materials and methods

### Cell culture and reagents

The human hepatocarcinoma cell lines, SMMC-7721 and Hep3B, were maintained in Dulbecco’s modified Eagle’s medium (DMEM) (GIBCO, Invitrogen) and supplemented with 10 % fetal bovine serum at 37 °C in a humidified 5 % CO_2_ incubator. Nutrient deprivation was carried out in Earle’s balanced salts (EBSS) medium (Sigma-Aldrich). 3-Methyladenine (3-MA), chloroquine (CQ), and *N*-acetyl-cysteine (NAC) were obtained from Sigma-Aldrich (Shanghai, China). Bevacizumab was obtained from Roche (Shanghai, China).

### Cell viability assay

The measurement of viable cell mass was assessed by a Cell Counting Kit (Dojin Laboratories, Kumamoto, Japan), as previously described [13].

### Western blot analysis

Western blot analysis was performed as described [14]. Antibodies used were specific for Beclin1, LC3, HIF-1α, and GAPDH (Epitomics, Inc.).

### Immunohistochemical analysis

Excised tumor tissue specimens from nude mice were formalin-fixed and embedded in paraffin. Sections 3–5 μm thick were cut (*n* = 4 tumor sections from each tumor), mounted, and stained with the following antibodies: rabbit anti-CD31 (1:50; Bioworld Technology), rabbit anti-PCNA (1:2,000; Bioworld Technology), rabbit anti-activated caspase-3 (1:1,000; Bioworld Technology), rabbit anti-Beclin1 (1:200; Novus Biologicals), rabbit anti-LC3 (1:200; Novus), mouse anti-HIF-1α (1:100; Santa Cruz), and goat anti-8 hydroxyguanosine antibody (1:400, Abcam). Horseradish peroxidase (HRP)-labeled goat anti-rabbit immunoglobulin and HRP-labeled goat anti-mouse immunoglobulin (Santa Cruz) were applied as secondary antibodies for 30 min at 37 °C, followed by the streptavidin–biotin complex method. Immunostaining was developed with DAKO Liquid DAB + Substrate–Chromogen System (Changdao BioTech), followed by counterstaining with hematoxylin. PCNA-positive nuclei were counted in five random fields per tumor (400×) and were expressed as a percentage of the total number of cells observed in each individual tumor. Activated caspase-3-positive cells were counted in five random fields per tumor (200×). Data were expressed as mean ± standard deviation (SD). Tumor tissue images were taken using the LEICA DM RXA2 microscope.

### TUNEL assay

Analysis of apoptotic cells in tumor tissue was performed by terminal deoxynucleotidyl transferase-mediated dUTP nickend labeling (TUNEL) staining using the apoptotic cell detection kit following the manufacturer’s directions (Merck Corp.). TUNEL-positive (apoptosis) cells had a pyknotic nucleus with dark green fluorescent staining. Each sample was observed at a magnification of 400×, and TUNEL-positive cells were calculated in five random fields per tumor.

### siRNA oligonucleotides and recombinant adenovirus

Stealth RNAiTM siRNA duplex oligoribonucleotides were used as siRNAs to human Beclin1 [14]. The production of recombinant adenovirus (Adsi-Beclin1) was performed as previously described [15]. The synthesized oligonucleotides against Beclin1 were: forward, 5′-GATCCCCCAGTTTGGCACAATCAATATTCAAGAGATATTGATTGTGCCAAACTGTTTTTA-3′ and reverse, 5′-AGCTTAAAAACAGTTTGGCACAATCAATATCTCTTGAATATTGATTGTGCCAAACTGGGG-3′.

### Transient transfections

GFP-LC3-expressing plasmids were transiently transfected into HCC cells as previously described [14].

### Cell apoptosis assay

Apoptosis detection by Annexin V/PI and DAPI staining were performed as described [14].

### ROS examination

To examine the accumulation of reactive oxygen species (ROS), cells were incubated with 10 μM 5- and-6-chloromethyl-20,70-dichlorodihydrofluorescein diacetate (CM-H_2_DCFDA/DCFH-DA; Invitrogen) for 30 min at 37 °C, respectively, followed by fluorescence microscopy.

### In vivo studies

To establish a hepatocarcinoma xenograft tumor model, SMMC7721 and Hep3B cells (2.0 × 10^7^ cells in 0.2 ml serum-free DMEM medium) were injected subcutaneously into the right back of male athymic BALB/c nu/nu mice (5-week-old). Tumor growth was monitored with electronic calipers using the formula: $\mathrm{volume}=a\times {b^2}/2$, where *a* was the width at the widest point of the tumor, and *b* was maximal width. When the tumors reached a mean tumor volume of 150–160 mm^3^, mice were randomly divided into five groups (each group had five mice) as follows: (a) control group (no treatment); (b) vehicle group (0.9 % sodium chloride solution or AdSi-blank); (c) bevacizumab group; (d) autophagy inhibition (chloroquine or AdSi-Beclin1); (e) combination group. Mice received intraperitoneal injections of 5 mg/kg bevacizumab or 60 mg/kg CQ in 100 μl of 0.9 % sodium chloride solution, or were treated with AdSi-Beclin1 or AdSi-blank virus by way of multiple-center intratumoral injections of 50 μl thrice weekly. All BALB/c nude mice were killed after 3 weeks of treatment.

### Statistical analysis

Values were expressed as mean ± SD. Statistical analysis between the two groups was calculated using Student’s *t* test, and analysis between multiple groups was calculated using the SPSS program, version 15.0. A *p* < 0.05 was considered statistically significant.

## Results

### Effects of bevacizumab on xenograft tumors and autophagy

**Fig. 1:**
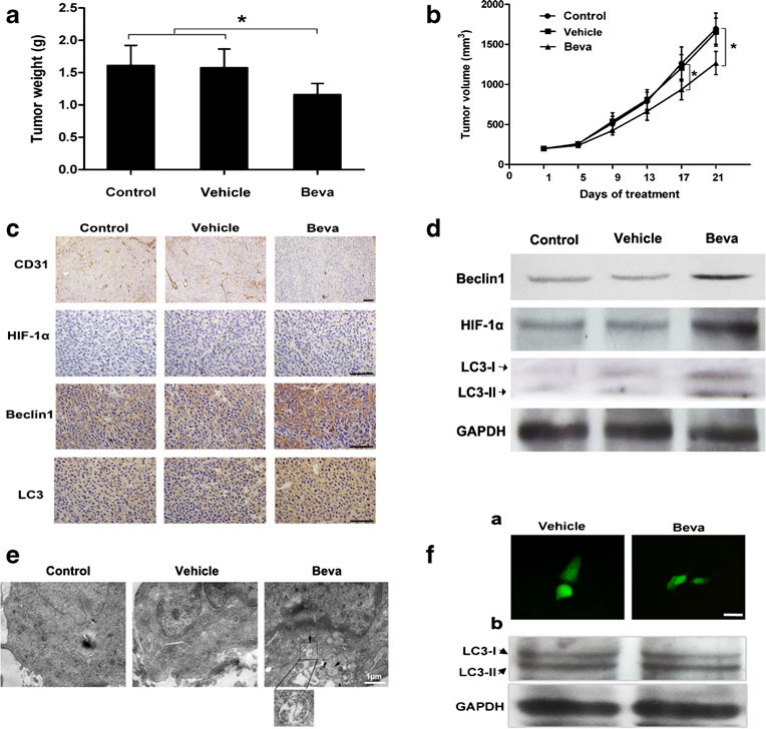
Bevacizumab inhibits tumor growth and induces autophagy in SMMC-7721 xenograft model. The tumor models are described in the “Materials and methods” section. **a** After 21 days of treatment, the tumor xenografts were excised, and tumor weights were measured. Data (mean ± SD) represent the mean of at least three independent experiments. **b** Tumor progression was evaluated by measuring tumor volume every 4 days. **c** Immunohistochemical staining of CD31, Beclin1, LC3, and HIF-1α. **d** Whole cell lysates of SMMC-7721 xenograft tumors were subjected to Western blot analysis for Beclin1, LC3, and HIF-1α. GAPDH expression was used as a loading control (scale *bar*, 100 μm)*. Beva*: bevacizumab. **e** Representative electron microscopic images are shown. An *arrow* indicates the autophagosome. **f** SMMC-7721 cells were transfected with a GFP-LC3 plasmid and were then treated with vehicle and bevacizumab (25 μg/ml) for 24 h (scale *bar*, 50 μm). Representative images are shown (**a**). Whole cell lysates were subject to Western blotting for LC3 (**b**)

To evaluate the effect of antiangiogenesis on xenograft tumor growth, bevacizumab was used as an antigiogentic agent. As shown in Fig. 1a, after 21 days of treatment, the mean SMMC7721 xenograft tumor weight of bevacizumab treatment group was significantly reduced compared with that of the control (1.16 ± 0.15 g versus 1.61 ± 0.28 g; *p* < 0.05) and vehicle (PBS) (1.16 ± 0.15 g versus 1.57 ± 0.26 g; *p* < 0.05) groups (Fig. 1a). The mean tumor volume of the bevacizumab group was also markedly reduced compared with that of the control (1,266.78 ± 145.77 mm^3^ versus 1,695.63 ± 194.74 mm^3^; *p* < 0.05) and vehicle (1,266.78 ± 145.77 mm^3^ versus 1,652.24 ± 175.86 mm^3^; *p* < 0.05) groups (Fig. 1b). In Hep3B xenograft tumors, similar results were observed (Supplementary Fig. 3a,3b). Moreover, CD31 immunohistochemical staining showed lower microvessel density in the bevacizumab-treated tumor (Fig. 1c). Additionally, the higher expression of HIF-1α in bevacizumab-treated tumors suggested that bevacizumab led to the enhancement of metabolic stress (Fig. 1c, d and Supplementary Fig. 3c).

Antiangiogenesis treatment leads to hypoxia and nutrient stress, both of which activate autophagy. Thus, we were interested in determining the effect of bevacizumab on autophagy in xenograft tumors. As shown in Fig. 1c and d, expression of Beclin1 and LC3 were upregulated in the bevacizumab group compared with the control and vehicle groups, which indicated activation of autophagy. Furthermore, electron microscopy analysis demonstrated an increase in autophagosomes in bevacizumab-treated tumors (Fig. 1e and Supplementary Fig. 3c). However, bevacizumab (25 μg/ml) could not activate autophagy in SMMC-7721 cells in vitro (Fig. 1f). Taken together, these results demonstrated that bevacizumab repressed tumor growth, reduced microvessel density, and induced autophagy in xenograft tumors.

### Inhibition of autophagy increases apoptosis of hepatocarcinoma cells during nutrient starvation and hypoxia in vitro

**Fig. 2:**
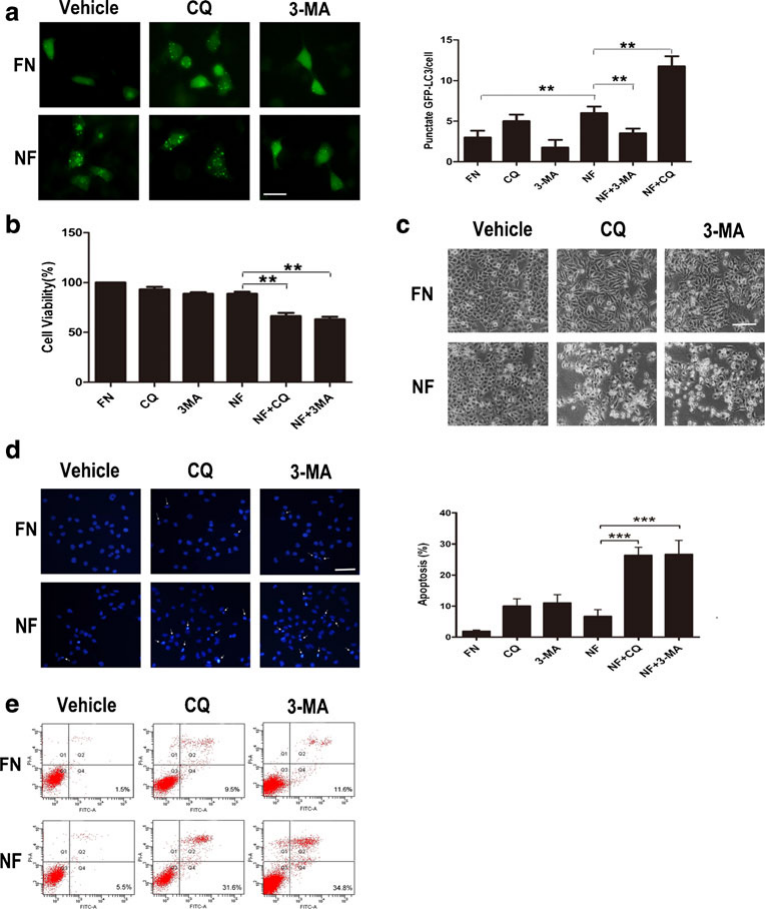
Autophagy inhibition by 3-MA or CQ promotes apoptosis of SMMC-7721 cells during nutrient starvation. SMMC-7721 cells were cultured in nutrient-starved medium and treated with CQ (20 mM) or 3-MA (10 mM) for 24 h. **a** After being transfected with GFP-tagged LC3 for 24 h, SMMC-7721cells were treated as mentioned above, followed by fluorescence microscopy (*left side*, scale *bar*, 50 μm). The *right side* is quantitative analysis of GFP-LC3 punctate dots/cell. **b** Cell viability was determined by a WST-8 assay. **c** Cell morphology is shown (scale *bar*, 50 μm). **d** Apoptotic cells were captured by DAPI staining of the condensed and fragmented nuclei. The *arrow* indicates apoptotic cells (*left side*, scale *bar*, 50 μm). The *right side* is quantitative analysis of apoptotic cells. **e** Analysis of Annexin-V and PI staining. The Annexin V^+^/PI^−^ or Annexin V^+^/PI^+^ cells were considered apoptotic cells. Data of three replicates are shown as means ± SD. *(*p* < 0.05), **(*p* < 0.01), ***(*p* < 0.001). *FN*: full nutrient, *NF*: nutrient-free

**Fig. 3:**
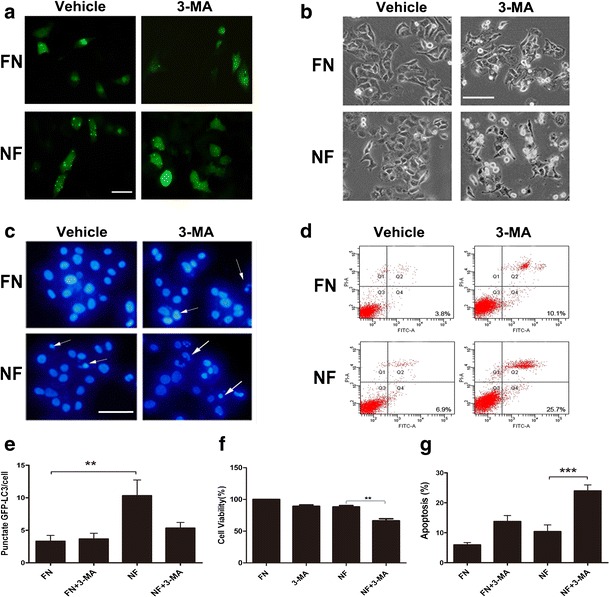
3-MA promotes the apoptosis of Hep3B cells during nutrient starvation. Hep3B cells were cultured in nutrient-starved medium and treated with 3-MA (10 mM) for 24 h. **a** After transfection with GFP-tagged LC3 for 24 h, Hep3B cells were treated as mentioned above, and images were taken under a fluorescence microscope(scale *bar*, 50 μm). **b** Cell morphology is shown (scale *bar*, 50 μm). **c** Apoptotic cells were captured by DAPI staining of the condensed and fragmented nuclei. The *arrow* indicates apoptotic cells (scale *bar*, 50 μm). **d** Analysis of Annexin-V and PI staining. Annexin V^+^/PI^−^ or Annexin V^+^/PI^+^ cells were considered apoptotic cells. **e** Quantitative analysis of GFP-LC3 punctate dots/cell of **a**. **f** Cell viability was determined by a WST-8 assay. (**g**) Quantitative analysis of apoptotic cells of **d**. Data of three replicates are shown as mean ± SD. **(*p* < 0.01), ***(*p* < 0.001). *FN*: full nutrient, *NF*: nutrient-free

Reducing tumor vasculature by antiangiogenic agents leads to nutrient starvation and hypoxia. These conditions of nutrient deficiency activate autophagy. To evaluate the effect of autophagy on nutrient deficiency in HCC cells, nutrient-deprived medium and hypoxia were used to mimic the nutrient-deficient condition, which tumor cells were exposed to in vivo during bevacizumab treatment. First, SMMC7721 cells, transfected with a GFP-LC3 plasmid, were cultured in nutrient-starved medium. As shown in Fig. 2a, nutrient-starved SMMC7721 cells had a significantly increased number of GFP-LC3 dots, an indicator of autophagy activation. This was enhanced by CQ treatment and attenuated by 3-MA. The results of WTS-8 assay showed that 3-MA or CQ treatment led to an increase in cell death (Fig. 2b). Cell morphology detection demonstrated that typical apoptotic changes appeared in 3-MA or CQ-treated nutrient-starved cells, including marked rounding, shrinkage, and detachment of cells from the culture dish (Fig. 2c). The results of DAPI and Annexin V/PI staining also demonstrated that treatment with autophagy inhibitor in nutrient-starved SMMC7721 cells induced significantly more apoptosis (Fig. 2d, e). The same results were also observed in Hep3B cells (Fig. 3). Taken together, these data suggest that inhibition of autophagy results in increased apoptosis in HCC cells during nutrient starvation.

To further confirm the role of the autophagic machinery, we used siRNA to inhibit the Beclin1 gene, which is essential for autophagosome generation [16]. As shown in Supplementary Fig. 1, silencing of Beclin1 markedly increased nutrient starvation-induced apoptosis in HCC cells. Together, these data strongly indicate that HCC cells resist nutrient starvation-induced apoptosis by activating autophagy.

**Fig. 4:**
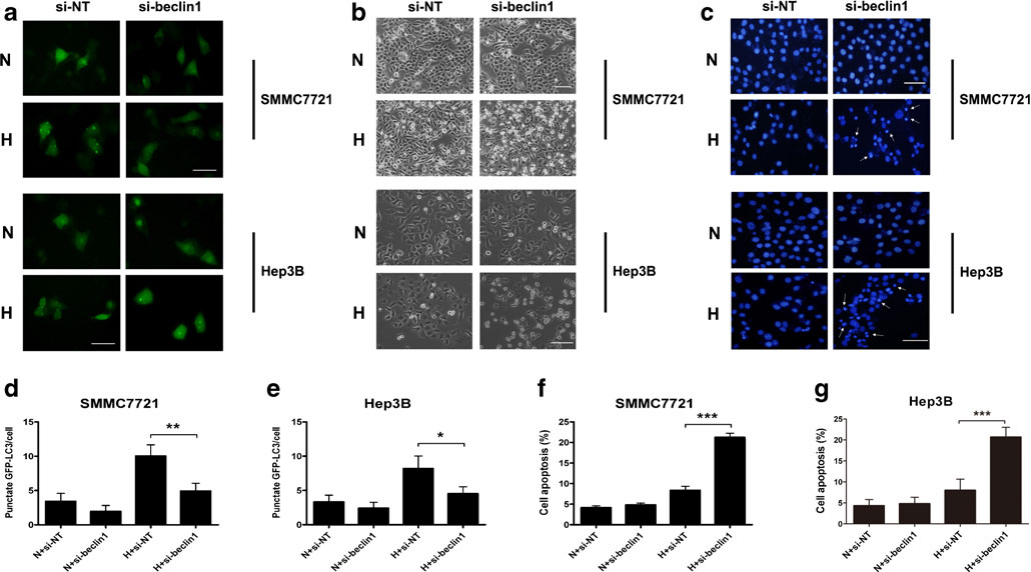
SiBeclin1 reduces cell viability and promotes apoptosis of HCC cells during hypoxic stress. After transfection with the siBeclin1 for 1 day, SMMC-7721 and Hep3B cells were cultured under normoxia (20 % O_2_) or hypoxia (1 % O_2_) for 36 h. **a** HCC cells were transfected with a GFP-tagged LC3 plasmid, and images were taken under a fluorescence microscope (scale *bar*, 50 μm). **b** Cell morphology was captured by a light microscope (scale *bar*, 50 μm). **c** Apoptotic cells were detected by DAPI staining. *Arrows* indicate the apoptotic cell (scale *bar*, 50 μm). **d**, **e** Quantitative analysis of GFP-LC3 punctate dots/cell of **a**. **f**, **g** Quantitative analysis of apoptotic cells of **c**. Data represent the mean of three independent experiments and are shown as mean ± SD. *(*p* < 0.05), **(*p* < 0.01), ***(*p* < 0.001). *si-NT*: no target siRNA. *N*: normoxia, *H*: hypoxia

To test the protective role of autophagy on HCC cells during hypoxia, SMMC7721 and Hep3B cells, transfected with either no-target siRNA or Beclin1 siRNA, were cultured under normoxia (20 % O_2_) or hypoxia (1 % O_2_) for 36 h. As shown in Fig. 4a, d, and e, hypoxic HCC cells showed an increase in the formation of GFP-LC3 dots, which was markedly inhibited by siBeclin1. Moreover, siBeclin1 treatment significantly increased apoptosis of hypoxic HCC cells (Fig. 4b, c, f, g). Thus, our data demonstrated that autophagy promoted HCC cell survival under hypoxic conditions.

### Inhibition of autophagy promotes the anticancer effects of bevacizumab in HCC xenograft tumors

**Fig. 5:**
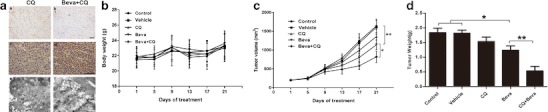
Therapeutic efficacy of bevacizumab was enhanced by CQ in hepatocarcinoma xenograft model. Tumor models were established. **a** SMMC-7721 tumor xenografts were treated with CQ or a combination of CQ and bevacizumab. Immunohistochemical staining for CD31 (**a** and **b**) and LC3 (**c** and **d**) was detected. Representative electron microscopic images of autophagosome (**e** and **f**) are shown. An *arrow* indicates the autophagosome (scale *bar*, 100 μm). **b** Body weights of all mice were measured every 4 days. **c** Tumor progression was evaluated by measuring tumor volume every 4 days. **d** After 21 days of treatment, mice were sacrificed, and tumor weights were measured. Data represent the mean of three independent experiments and are shown as mean ± SD. *(*p* < 0.05), **(*p* < 0.01). *Beva*: bevacizumab

To investigate the in vivo efficacy of the combined treatment of an autophagy inhibitor and angiogenesis inhibitor in hepatocarcinoma, a SMMC-7721 hepatocarcinoma xenograft tumor model was established in nude mice. As shown in Fig. 5a, the increased accumulation of LC3 and formation of the autophagosome were observed in the CQ and bevacizumab combination treatment group. These data suggest that CQ can successfully inhibit autophagy in vivo. The results of CD31 staining also suggested that CQ had no effect on angiogensis in xenograft tumors. Furthermore, there were no significant differences in the body weight of nude mice between groups (Fig. 5b). After 21 days of treatment, the mean tumor volume of the combination group was significantly reduced compared with the bevacizumab group (818.68 ± 152.52 mm^3^ versus 1,138.41 ± 129.14 mm^3^; *p* < 0.05; Fig. 5c). Meanwhile, compared with the bevacizumab group, the mean tumor weight of the combination group was significantly reduced by 27.46 % (*p* < 0.05; Fig. 5d). The same results were also observed in Hep3B xenograft tumors (Supplementary Fig. 3a,3b). Together, these results indicate that the combined treatment of CQ and bevacizumab can significantly inhibit tumor growth in vivo.

### Combination bevacizumab and chloroquine treatment inhibits cell proliferation and increases intratumoral apoptosis in HCC xenograft tumors

**Fig. 6:**
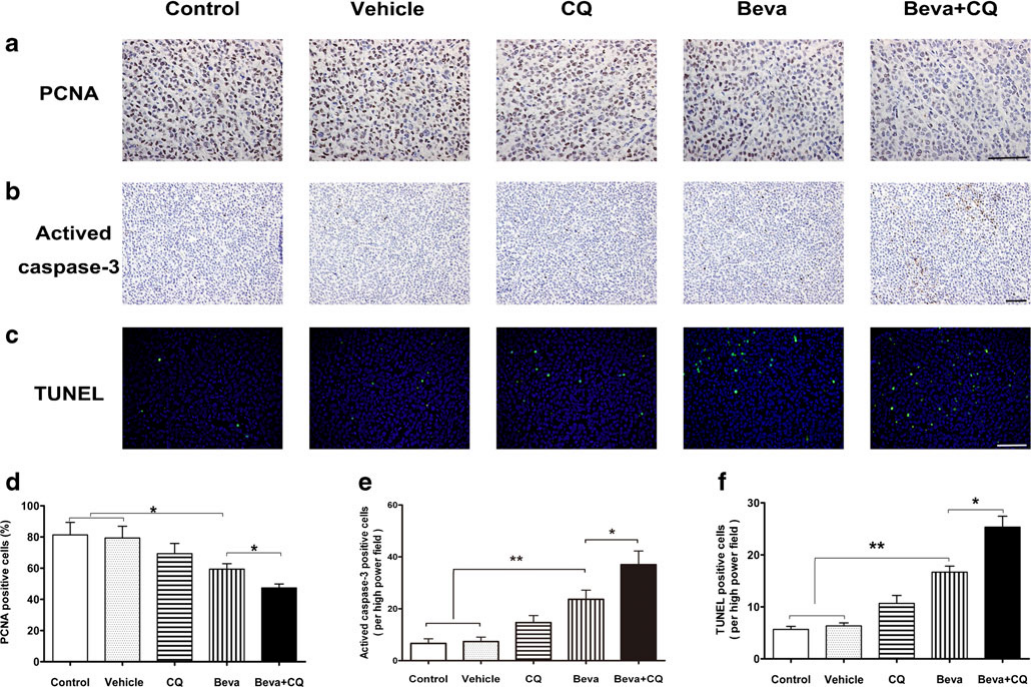
Combination treatment of bevacizumab and CQ leads to reduced cell proliferation and increased apoptosis in a hepatocarcinoma xenograft tumor model. **a**, **b** Immunohistochemical staining of representative tumor tissue samples with PCNA and activated caspase-3 antibodies(scale *bar*, 100 μm). **c** Apoptosis of tumor tissues was measured by TUNEL assays; *green* nucleus shows TUNEL-positive cells(scale *bar*, 100 μm). **d**, **e** Quantitative analysis of proliferative and caspase-3-activated cells. **f** Quantitative analysis of TUNEL-positive cells. Data represent three independent experiments shown as mean ± SD. *(*p* < 0.05), **(*p* < 0.01). *Beva*: bevacizumab

Metabolic stress has been reported to have the ability to induce apoptosis [17]. To elucidate the potential mechanism underlying the tumor-suppressive function of combined bevacizumab and CQ treatment, we examined the proliferation and apoptosis of xenograft tumor cells. Proliferating cell nuclear antigen (PCNA) is a widely used marker of cell proliferation. As shown in Fig. 6a and d, PCNA expression was significantly reduced in the bevacizumab group compared with the control and vehicle groups. In addition, a combination of bevacizumab and CQ treatment further reduced PCNA expression when compared with treatment with bevacizumab alone (47.35 ± 2.51 % versus 59.26 ± 3.52 %; *p* < 0.05). To detect tumor cell apoptosis, activated caspase-3 immunostaining and TUNEL assay were performed. As shown in Fig. 6b and e, activated caspase-3 expression was significantly upregulated in the combined bevacizumab and CQ treatment group compared with the bevacizumab treatment group (37.67 ± 3.21 % versus 23.32 ± 2.88 %; *p* < 0.05). These results were further confirmed by the TUNEL assay, which showed that combination of bevacizumab and chloroquine treatment induced more apoptosis in tumor cells than bevacizumab treatment alone (25.45 ± 2.08 % versus 16.67 ± 1.15 %; *p* < 0.05, Fig. 6c, f). Similarly, in Hep3B xenograft tumor model, combined treatment with bevacizumab and chloroquine also impaired tumor cell proliferation and increased intratumoral apoptosis (Supplementary Fig. 3c–3e). These results suggest that impaired cell proliferation and increased cell apoptosis contribute to the tumor-suppressive function of bevacizumab and CQ combination treatment.

### ROS generation is enhanced and contributes to cell death in nutrient-deficient HCC cells during autophagy inhibition

**Fig. 7:**
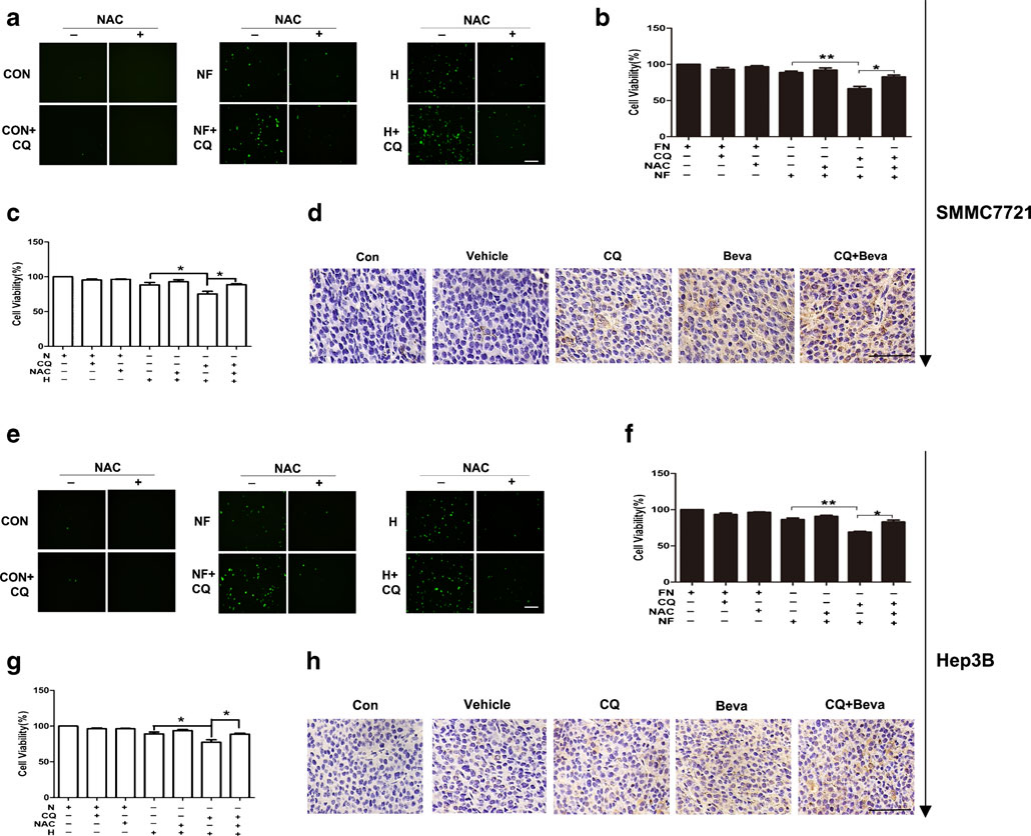
ROS generation is augmented and contributes to cell death in nutrient-deficient HCC cells when autophagy is inhibited. SMMC-7721 and Hep3B cells were incubated in nutrient-starved medium for 24 h or hypoxia for 36 h with 10 μM CQ. **a**, **e** Cellular ROS generation was determined using DCF-DA staining (scale *bar*, 100 μm). **b**, **c**, **f**, **g** Cells were pretreated with 10 mM NAC for 2 h; then cells were incubated in EBSS medium or hypoxia alone or with 10 μM CQ for the indicated time. Cell viability was determined by a WST-8 assay. Data shown are mean ± SD from at least three independent experiments. **d**, **h** Immunohistochemical staining of 8-OhdG (scale *bar*, 100 μm). *FN*: full nutrient, *NF*: nutrient-free, *N*: normoxia, *H*: hypoxia

Metabolic stress causes ROS accumulation and increased ROS leads to cell death. To determine the role of ROS in cell death of nutrient-deficient HCC cells after autophagy inhibition, we first detected whether autophagy could modulate ROS generation in nutrient-deficient cells. Intracellular levels of ROS in HCC cells following treatment with CQ, nutrient-starved medium, hypoxia, or the combination for indicated time (nutrient starvation for 24 h; hypoxia for 36 h) were examined. In both SMMC-7721 and Hep3B cell lines, there were marked increases in the ROS levels after treatment with CQ and nutrient starvation or hypoxia when compared with cells under nutrient starvation, hypoxia, or CQ treatment alone (Fig. 7a,e). To evaluate whether enhanced ROS levels may contribute to the cell death of nutrient-deficient HCC cells with autophagy inhibition, we applied the antioxidant NAC to eliminate ROS. SMMC-7721 and Hep3B cells pretreated with NAC displayed significantly reduced cell death with CQ and nutrient starvation or hypoxia combined treatment (Fig. 7b, c, f, and g). Thus, increased ROS levels have an important role in the induction of cell death by nutrient deficiency in combination with autophagy inhibitor. We also examined the immunostaining of 8-hydroxydeoxyguanosine (8-OHdG) in xenograft tumor tissue, as 8-OHdG is an indicator of DNA oxidative damage. As shown in Fig. 7d and h, more 8-OHdG-positive cells were observed in the bevacizumab and CQ cotreatment group. Together, these results suggest that autophagy inhibition enhances metabolic stress-induced oxidative damage, which contributes to the death of nutrient-starved HCC cells.

## Discussion

We report here that bevacizumab treatment inhibits the growth of xenograft tumors and activates autophagy by reducing the formation of new blood vessels. The combination of autophagy inhibitor and bevacizumab treatment further inhibited HCC xenograft tumor growth, with enhanced apoptosis and impaired cell proliferation. In addition, autophagy inhibition led to enhanced ROS generation in nutrient-deficient HCC cells in vitro and increased DNA oxidative damage in vivo. Our results suggest that autophagy modulates ROS generation and contributes to cell survival under metabolic stress; thus, inhibition of autophagy may be a novel way to increase the efficicacy of antiangiogenic agents in treating HCC.

Antiangiogenic strategy deprives tumors of their blood supply by reducing the tumor vasculature, which leads to the induction of necrosis or apoptosis in tumor cells, ultimately inducing tumor regression [18]. In this study, bevacizumab treatment inhibited the growth of tumor xenografts and activated autophagy by reducing blood vessel generation. However, bevacizumab treatment was not able to induce autophagy in HCC cells in vitro. Autophagy can be activated by metabolic stress[19]. Thus, the upregulation of autophagy during bevacizumab treatment is a response of xenograft tumor cells to metabolic stress.

Under nutrient deficiency, autophagy is induced and promotes tumor cell survival [20, 21]. Consistent with this, we demonstrated here that autophagy protected HCC cells from nutrient starvation and hypoxia-induced apoptosis in vitro. Interestingly, early reports have shown that autophagy activated by hypoxia can mediate the tolerance of hepatocellular carcinoma cells to nutrient deprivation [22]. Autophagy promotes cell survival in various ways, such as providing energy substrates, mitigating accumulated ROS, and reducing damaged organelles [20]. In fact, in the early or later stages of tumor progression, autophagy is activated in metabolically stressed tumor regions [23]. These findings suggest that maintaining cell survival by autophagy in unfavorable conditions is essential for tumor development. Thus, we suspect that inhibition of autophagy may be an effective way of improving antiangiogenic strategy in vivo. In this study, we observed that the combined treatment of bevacizumab and CQ led to dramatic tumor repression. These results indicate that inhibition of autophagy impairs the survival mechanism of tumors and intensifies metabolic stress, consequently leading to tumor inhibition. Indeed, previous studies have shown that endothelial cells initiate autophagy as a survival mechanism against angiogenesis inhibitor-induced apoptosis [24, 25]. These findings further strengthen our hypothesis that autophagy inhibition can enhance the therapeutic efficacy of antiangiogenic strategy. In this study, we showed that the enhancement of apoptosis and impairment of cell proliferation are most likely the mechanisms underlying the antitumor effects of combined bevacizumab and CQ treatment. Meanwhile, inhibition of autophagy may impair its function of preserving organelle required for cell growth [20] and render cell cycle arrest [26].

It was suggested that low concentrations of ROS promoted cell proliferation [27], but high ROS concentrations lead to cell death [28]. Therefore, modulation of intracellular ROS levels is critical for cancer cell survival, as metabolic stress causes ROS accumulation [29, 30]. In this study, we observed a modest increase in cellular ROS generation in nutrient-starved and hypoxic HCC cells. However, the combination of CQ treatment and nutrient starvation or hypoxia resulted in a marked increase in ROS generation. Increased levels of ROS contributed to cell death in nutrient-starved or hypoxic HCC cells when autophagy was inhibited, but treatment with the NAC antioxidant markedly reduced this phenonemon. Consistent with our findings, a recent study suggested that induction of autophagy could decrease ROS levels and moderate ROS-induced cell death [30]. The removal of oxidatively damaged organelles and proteins by autophagy provides a second level of protection against oxidative stress [31]. However, the interaction between ROS and autophagy is complex. ROS, especially mitochondrial ROS, serve as signaling molecules in inducing autophagy [32, 33].

Although angiogenesis inhibitors were considered as desirable anticancer agents, it was found that most tumors were still resistant to antiangiogenesis [34, 35]. The mechanisms of resistance to antiangiogenesis are still unknown [34, 35]. In this study, we provide compelling data suggesting that inhibition of autophagy can enhance the tumor-suppressive function of bevacizumab. Thus, activation of autophagy may be involved in resistance to antiangiogenic strategies. Autophagy may help tumor cells mitigate metabolic stress and promote cell survival during antiangiogenesis, which may provide tumor cells with a greater change to develop other ways to resist antiangiogentic therapy. In conclusion, we show that inhibition of autophagy may be a novel way to increase the efficicacy of antiangiogenic agents in the treatment of HCC.

## Electronic supplementary material

Below is the link to the electronic supplementary material.

ESM 1 (PDF 441 kb)
