# Supplementary material for: Inhibition of autophagy enhances anticancer effects of bevacizumab in hepatocarcinoma
Source: J Mol Med (Berl). 2012 Oct 10;91(4):473–83. doi: 10.1007/s00109-012-0966-0 (PMC3611041; doi:10.1007/s00109-012-0966-0)
Supplement: Supplementary file 1 — (PDF 441 kb) [file 109_2012_966_MOESM1_ESM.pdf]

## Figure legends for supplementary data:

### Supplementary Figure 1.

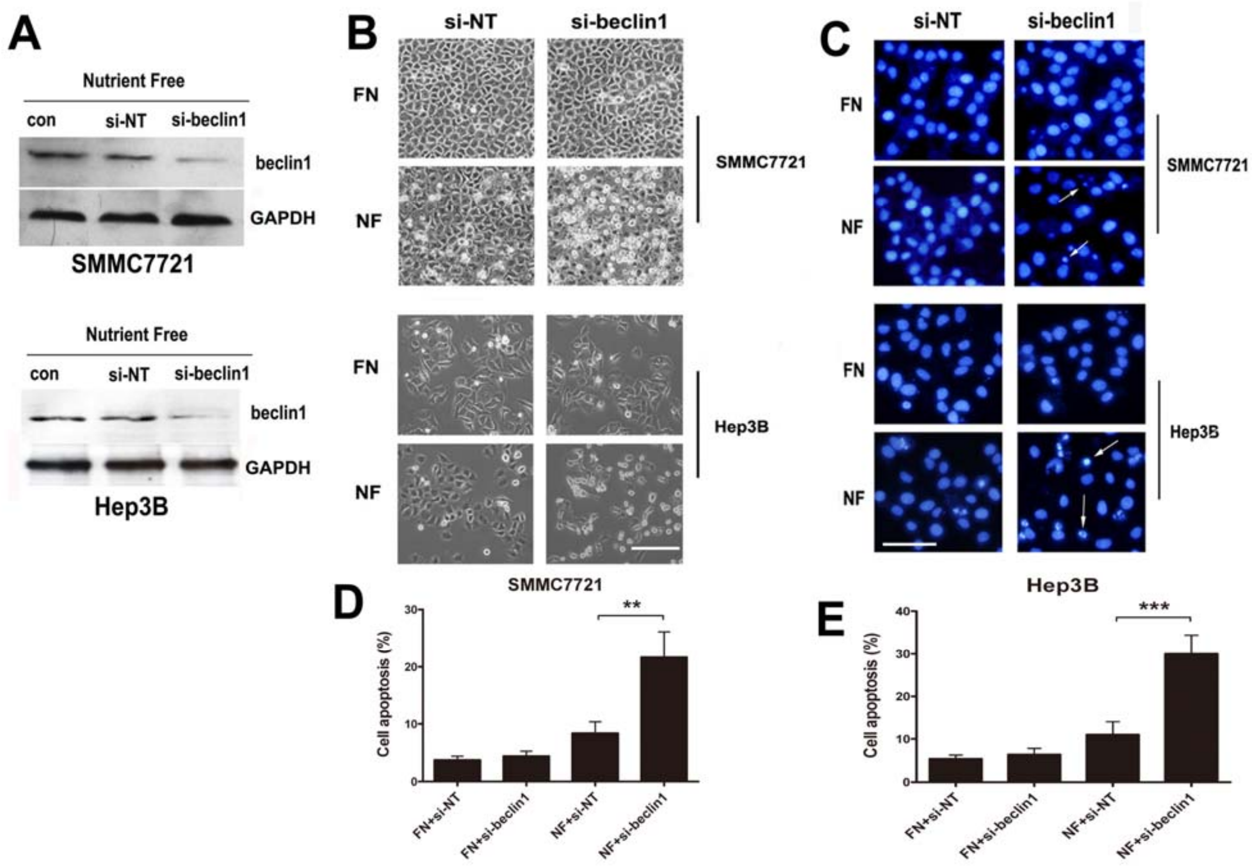


**Si-Beclin1 reduces the cell viability and promotes apoptosis of SMMC7721 and Hep3B cells during nutrient starvation**

SMMC7721and Hep3B cells were transfected with either notarget siRNA or the Beclin1 siRNA, one day later, cells were cultured in nutrient-starved (EBSS medium) or nutrient-containing medium for 24hours**. (A)** Whole cell lysates were subjected to western blot analysis for beclin1. si-NT, RNAi no target. **(B)** The morphology of the cells was detected by a light microscope (scale bar:50μm). **(C)** The fluorescent microscopic pictures of apoptotic cells were captured by DAPI staining of the condensed and fragmented nuclei. Arrow indicated apoptotic cell (scale bar:50μm).

**(D** and **E)** Quantitative analysis of apoptosis cells of **(C)**. Data represent the means of three independent experiments as means ±SD. **($p < 0.01$), ***($p < 0.001$) FN: full nutrient, NF: nutrient free, si-NT: no target siRNA.

**Supplementary figure.2:**

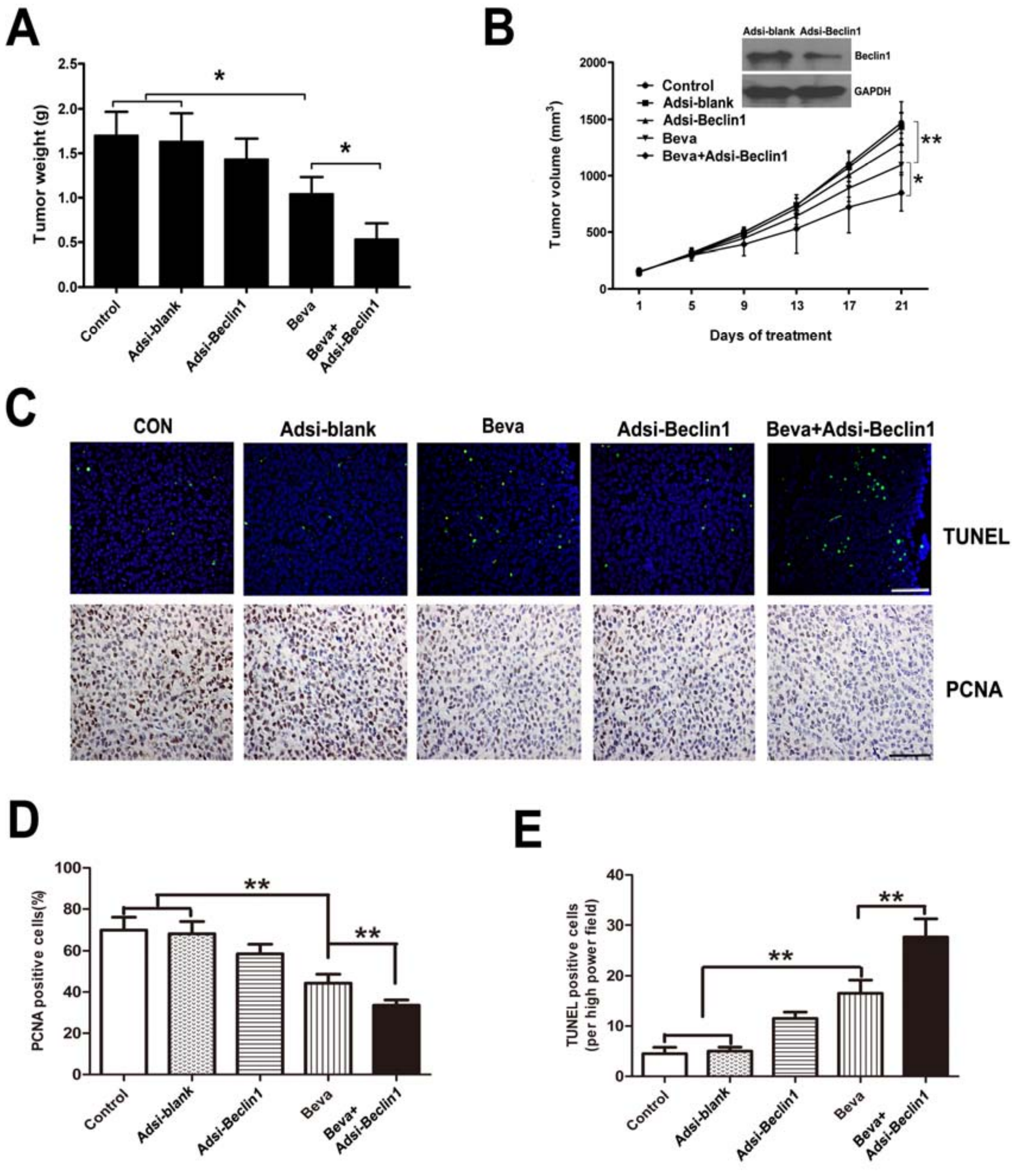


## Si-Beclin1 enhances therapeutic efficacy of bevacizumab in SMMC7721 hepatocarcinoma xenograft model

SMMC7721 HCC xenografted tumor models were established in BALB/c nu/nu mice, When the tumors reached a mean tumor volume of 150–160 mm3, mice were divided into five groups (n=5 for each group): control, Ad- si-blank, Ad-si-beclin1, bevacizumab (5 mg/kg), combination bevacizumab and si-beclin1. Ad-si-beclin1 and

Ad- si-blank (1x 109/50 ul/time, separately) were directly injected into the tumor in situ. Bevacizumab was injected into the abdominal cavity. All kinds of group received i.p. injections thrice weekly. (A) After 21 days treatment, mice were sacrificed, the tumor xenografts were excised and the tumor weights were measured. (B) Progression of tumor was evaluated by measurement of tumour volume every four day. (C-upper) Apoptosis of tumor tissues in different groups were measured by TUNEL assays, green nucleus shows the TUNEL positive cells. (C-lower) IHC staining of representative tumor tissue samples with PCNA antibodies (scale bar:100μm). (D) Quantitative analysis of tumor cells proliferation. (E) Quantitative analysis of TUNEL positive cells. Data represent the means of three independent experiments as means ±SD. *($p < 0.05$), ** ($p < 0.01$). Beva: bevacizumab,

**Supplementary figure.3:**

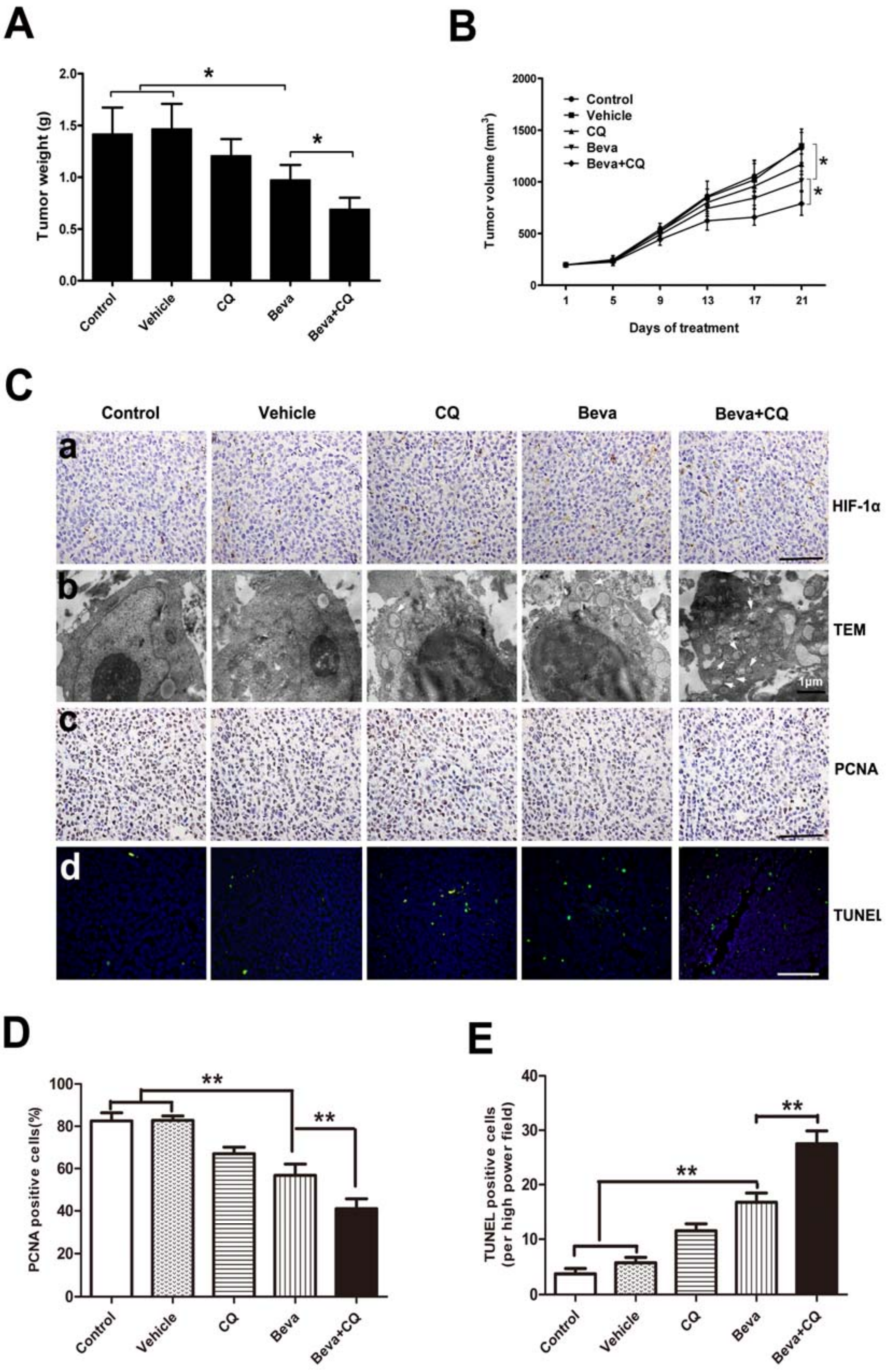

**Inhibition of autophagy by CQ enhances therapeutic efficacy of bevacizumab in Hep3B hepatocarcinoma xenograft model**

Hep3B HCC xenografted tumor models were established in BALB/c nu/nu mice, When the tumors reached a mean tumor volume of 150–160 mm3, mice were divided into five groups (n=5 for each group): control, vehcle (100µl of 0.9% sodium chloride solution), CQ (60mg/kg), bevacizumab (5 mg/kg), combination bevacizumab and CQ. Vehicle, bevacizumab, CQ and combination groups received i.p. injections thrice weekly. (A) After 21days treatment, mice were sacrificed, the tumor xenografts were excised and the tumor weights were measured. (B) Tumour progression of tumor was evaluated by measurement of tumour volume every four day. (C a and b) IHC staining of representative tumor tissue samples with HIF-1α and PCNA antibodies. (C c) Representative electron microscopic images of autophagosome (arrow indicated) were detected by TEM (transmission electron microscopy) and showed. (C d) Apoptosis of tumor tissues in different groups were measured by TUNEL assays, green nucleus shows the TUNEL positive cells (scale bar:100µm). (D) Quantitative analysis of tumor cells proliferation. (E) Quantitative analysis of TUNEL positive cells. Data represent the means of three independent experiments as means ±SD. *($p < 0.05$), ** ($p < 0.01$). Beva: bevacizumab
